# Constructing an Ensemble Model and Niche Comparison for the Management Planning of Eucalyptus Longhorned Borer *Phoracantha semipunctata* under Climate Change

**DOI:** 10.3390/insects14010084

**Published:** 2023-01-13

**Authors:** Haoxiang Zhao, Xiaoqing Xian, Te Liang, Fanghao Wan, Juan Shi, Wanxue Liu

**Affiliations:** 1 The College of Forestry, Beijing Forestry University, Beijing 100193, China; 2 State Key Laboratory for Biology of Plant Diseases and Insect Pests, Institute of Plant Protection, Chinese Academy of Agricultural Science, Beijing 100193, China

**Keywords:** invasive alien forest pest, *Phoracantha semipunctata*, potential geographical distribution, niche dynamic, distribution centroid

## Abstract

**Simple Summary:**

*Phoracantha semipunctata* is the worst forestry pest worldwide. It has spread to more than 30 countries or regions where eucalyptus species have been planted, damaging the eucalyptus species, thereby causing huge economic losses. It was also the quarantine pest for the European Plant Protection Organization and China. In our study, based on 865 global occurrence records of *P. semipunctata* and 10 environmental variables, the potential global geographical distributions of *P. semipunctata* were predicted by the ensemble model. Our findings showed that the potential global geographical distributions of *P. semipunctata* under the current climate are distributed in the six continents except for Antarctica worldwide. Future climate changes will decrease the suitable area for *P. semipunctata* in the 2030s and 2050s. *Phoracantha semipunctata* has largely conserved its niche in the invasive areas. More attention should be paid to early warning signs and monitoring of *P. semipunctata* in potentially invasive areas.

**Abstract:**

*Phoracantha semipunctata* is a destructive invasive alien forest pest worldwide. It primarily damages the eucalyptus via adults, affecting almost all parts of the eucalyptus. Its larvae develop in almost all major tissues of the plant. *Phoracantha semipunctata* spreads both via the migration of adults and global trade in intercontinental translocation. Currently, this pest has spread to six continents worldwide, except Antarctica, resulting in substantial economic losses. Based on global occurrence data and environmental variables, the potential global geographical distribution of *P. semipunctata* was predicted using an ensemble model. The centroid shift, overlap, unfilling, and expansion scheme were selected to assess niche dynamics during the global invasion process. Our results indicated that the AUC and TSS values of the ensemble model were 0.993 and 0.917, respectively, indicating the high prediction accuracy of the model. The distribution pattern of *P. semipunctata* is primarily attributed to the temperature seasonality (bio4), mean temperature of the warmest quarter (bio10), and human influence index variables. The potential geographical distribution of *P. semipunctata* is primarily in western and southwestern Asia, western Europe, western and southern North America, southern South America, southern Africa, and eastern and southern Oceania. The potential geographical distribution of *P. semipunctata* showed a downward trend in the 2030s and the 2050s. The distribution centroid showed a general tendency to shift southward from the near-current to future climate. *Phoracantha semipunctata* has largely conserved its niche during the global invasion process. More attention should be paid to the early warning, prevention, and control of *P. semipunctata* in the countries and regions where it has not yet become invasive.

## 1. Introduction

Biological invasions by invasive alien forest pests (IAFPs) pose a significant threat to forests by damaging or killing trees, resulting in global-scale ecological and economic impacts [1,2,3]. For example, IAFPs damage plantations and certain valuable trees and reduce their regeneration, such as in pine, elm, and eucalyptus [1,4,5]. IAFPs destroy approximately 4000 km^2^ of forest per year in Canada and approximately 28,000 km^2^ in China [6,7]. The economic impact of IAFPs in terms of lost timber value is likely to be $150 million in the United States [8]. Rapid climate warming has altered the distribution pattern of IAFPs and hosts in the Anthropocene, which might facilitate their global invasion [9,10]. Climate suitability has an important impact on the degree of invasion and spread risk posed by IAFPs [5]. Based on the “climate match hypothesis,” the establishment of species populations is more likely to succeed in areas with environmental conditions that are similar to those in native areas [11]. Nevertheless, a few previous studies have revealed that some species might occupy new environmental conditions in invasive areas by shifting their native niche, which might significantly affect invasion and spread risk [12]. Human-mediated global trade and long-distance transportation networks have resulted in the intercontinental translocation of IAFPs over the last few decades, exacerbating global invasion and spreading risk [4,13]. Consequently, the management planning of IAFPs that considers the impact of environmental suitability, human influence, and niche dynamics is pivotal for mitigating the negative impacts in the Anthropocene.

*Phoracantha semipunctata* (Fabricius) 1775 (Coleoptera: Cerambycidae), known as the eucalyptus longhorned borer, is native to Australia and has spread to more than 30 countries or regions where eucalyptus species have been planted [14,15]. The movement and dispersal of *P. semipunctata* primarily occurred via adult migration in the local zones and human-mediated transport of eucalyptus wood in intercontinental translocation [16,17]. *Phoracantha semipunctata* was first reported in South Africa as an introduced insect in 1906 [18]. When eucalyptus species were used to establish plantations in countries with Mediterranean climates until the late 20th century, the IAFP widely spread via the transport of eucalyptus species [18]. *Phoracantha semipunctata* poses a great threat to the eucalyptus plantation industry and the local economy in the invasive zone. The eucalyptus species that was attacked by *P. semipunctata* showed some dead branches with yellowing and wilting leaves, and the larval tunnels were filled with compressed frass, producing characteristically long, narrow lesions with dark exudation [18].

The pest caused an estimated loss of 2 million trees, representing approximately 1700 km^2^ of eucalyptus plantations in 1981 in Morocco [19]. The average tree mortality due to *P. semipunctata* was estimated at 2.23% to 3.88% during 1981–1983 in the Spanish province of Huelva, corresponding to a loss of 62.07 km^2^ [20]. *Phoracantha semipunctata* is an A2 quarantine pest for the European Plant Protection Organization (EPPO) [21] and is a quarantine pest in China. Nevertheless, there are currently no findings on the potential geographical distribution and niche dynamics of *P. semipunctata* under global climate change. These findings can help in management planning and establishing sustainable prevention networks.

Species distribution models (SDMs) are becoming increasingly powerful tools. SDMs primarily include (1) correlative models, such as artificial neural networks (ANN), categorical regression tree analysis (CTA), gradient boosting model (GBM), generalized linear model (GLM), maximum entropy model (MaxEnt), random forest (RF), surface range envelope (SRE), and flexible discriminant analysis (FDA), which are based on the statistical relationships between known species occurrence data and environmental variables [22], and (2) mechanistic models, such as the CLIMEX and insect life cycle model (ILCM), which are based on eco-physiological information [23]. SDMs are widely used to assess the risk of invasive alien species [24]. Nevertheless, single models might carry some uncertainties in the occurrence, environmental data, and model structure [25]. The ensemble model (EM) has shown superior predictive performance to single models in many cases, based on the combination of units of single models [26,27]. Consequently, using EM to model the potential geographical distribution of invasive alien species has become a significant measure for management planning.

In the present study, we are the first to estimate the distribution pattern of *P. semipunctata* using EM and to assess niche dynamics worldwide. Specifically, we aim to address three issues. First, we predicted the potential geographical distribution of *P. semipunctata* under current and future climates and assessed their changes. Second, we expected that established populations of *P. semipunctata* would occupy similar environmental niches in the invasive area to those of the native area, which should be consistently supported by the consensus of multiple tests performed with different metrics. Finally, we identified the significant environmental variables affecting the distribution of *P. semipunctata* worldwide.

## 2. Materials and Methods

### 2.1. Species Occurrence Data

**Figure 1:**
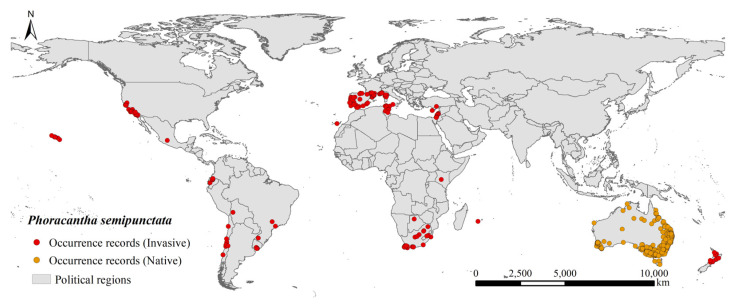
Invasive and native occurrence records of *Phoracantha semipunctata* worldwide.

In the present study, 1650 occurrence records were collected for *P. semipunctata* from 4 sources. A total of 1041 occurrence records were downloaded from the Global Biodiversity Information Facility [28]; 11 occurrence records were downloaded from the Barcode of Life Data Systems (BOLD, http://www.boldsystems.org/, accessed on 22 November 2022); 551 occurrence records were downloaded from Atlas of Living Australia (ALA, https://www.ala.org.au/, accessed 22 November 2022); and 47 occurrence records were obtained from publications in the Web of Science [29,30,31]. To avoid the effects of occurrence record bias and spatial autocorrelation on the accuracy of the results of model prediction [32], duplicate records **and** the occurrence records assigned to capital cities based on the centroids of the provinces were removed. Second, we selected ENMTools to ensure that only one occurrence record was present in each 5 km^2^ raster [33]. Ultimately, 865 global occurrence records were retained (Figure 1). 

### 2.2. Environmental Data

Environmental data were obtained from publicly available online databases. The near-current climate (1970–2000) and altitude data at a spatial resolution of 2.5 arc min were sourced from WorldClim v. 2.1 [34]. The future climate data for the 2030s (2020–2040) and 2050s (2041–2060) at a spatial resolution of 2.5 arc min were from WorldClim v. 2.1, including three shared socioeconomic pathways (SSP1-2.6, SSP2-4.5, and SSP5-8.5), which were based on the BCC-CSM2-MR climate system model. The near-current and future climate data include 19 bioclimatic variables (Table S1). The human influence index (HII) was downloaded from the Global Human Influence Index (Geographic) v2 (1995–2004), at a spatial resolution of 30 s. Finally, ArcGIS (version 10.8) was used to unify the spatial resolution of all environmental variables at 2.5 arc min.

The high multivariate collinearity of the environmental data can negatively influence the accuracy of SDM predictions. Consequently, ENMTools was used to eliminate the multivariate collinearity of environmental data (Figure S1) [33]. Finally, for any two highly correlated environmental variables (|r| > 0.8), the important bioclimatic variable was retained. Finally, we retained eight bioclimatic variables, altitude, and HII for modeling (Table S2).

### 2.3. Modeling Approach

Eight SDMs and an EM were used to model the potential global geographical distribution of *P. semipunctata*. The eight single SDMs included ANN, CTA, FDA, GBM, GLM, MaxEnt, RF, and SRE. In the modeling process, we selected 75% of the occurrence records of *P. semipunctata* as the training dataset and the remaining 25% as the testing dataset. 1000 pseudo-absence samples were randomly generated. Default settings were used for the other modeling parameters. We repeated the modeling process 10 times to eliminate the uncertainty of the SDMs. Ultimately, 80 single SDMs were constructed. The area under the receiver operating characteristic (ROC) curve (AUC) and true skill statistic (TSS) values were used as evaluation indices for the accuracy of the SDMs [35,36]. The values of AUC and TSS ranged from 0 to 1, and values of AUC and TSS closer to 1 indicated a better-performing SDM. The top three SDMs with the highest mean values of AUC and TSS were used to construct an EM. 

Based on the maximizing TSS value, which was used to transform the continuous values into binary in the EM results, the potential geographical distribution of *P. semipunctata* was classified into four types: highly suitable (600 ≤ *p* ≤ 1,000), moderately suitable (400 ≤ *p* < 600), lowly suitable (194 ≤ *p* < 400), and unsuitable (0 ≤ *p* < 194). The maximizing TSS value was also used to divide the potential geographical distribution of *P. semipunctata* into the suitable area (≥194) and unsuitable area (<194), thereby the species distribution model tool in ArcGIS (version 10.8) was used to analyze the shift of suitable area centroids of *P. semipunctata* under near-current and future climates.

### 2.4. Niche Measurement

The ecological niches of *P. semipunctata* between native (Australia) and invasive areas were compared using the ecospat package in R version 4.2 [37](Di Cola et al., 2017). Ten available environmental variables (altitude, HII, bio2, bio4, bio10, bio12, bio15, bio17, bio18, and bio19) were used to perform PCA-env and generate the predicted niche occupancy profiles. Niche overlap was calculated using Schoener’s ***D*** [38], which ranges from 0 (no overlap) to 1 (complete overlap). The ecospat package divides the niches of native and invasive areas into three components, including the centroid shift, overlap, unfilling, and expansion (COUE) [37].

The niche equivalency test was selected to calculate whether the niche overlap between the native and invasive areas was constant and was repeated 100 times. If the observed D value is not within 95% of the simulated values (*p* < 0.05), the null hypothesis of niche equivalency can be rejected, indicating that the niche in the invasive area is not equivalent to that in the native area [39]. Niche similarity tests were run in both directions (Australia → China) with 100 repetitions, which were selected to address the similarity between native and invasive areas. The significant niche similarity test results (*p* < 0.05) indicated that the observed niche overlap was more similar to each other than expected by chance [39].

## 3. Results

### 3.1. Model Performance

**Figure 2:**
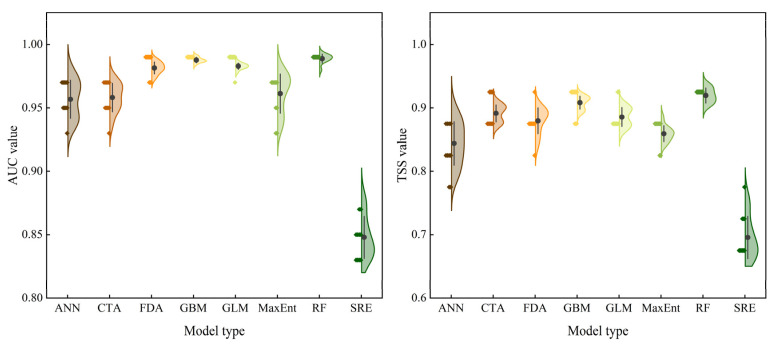
Evaluation indices of species distribution models (ROC: receiver operating characteristic, TSS: true skill statistic, ANN: artificial neural network, CTA: categorical regression tree analysis, GBM: gradient boosting model, GLM: generalized linear model, MaxEnt: maximum entropy model, RF: random forest, SRE: surface range envelope, and FDA: flexible discriminant analysis).

The mean AUC values for ANN, CTA, FDA, GBM, GLM, MaxEnt, RF, and SRE were 0.957, 0.958, 0.982, 0.988, 0.983, 0.961, 0.989, and 0.848, respectively (Figure 2). The mean TSS values for ANN, CTA, FDA, GBM, GLM, MaxEnt, RF, and SRE were 0.844, 0.891, 0.880, 0.908, 0.886, 0.859, 0.920, and 0.696, respectively (Figure 2). Based on the mean values of the AUC and TSS of the eight SDMs, we selected RF, GLM, and GBM to construct an EM. The mean AUC and TSS values for EM were 0.993 and 0.917, respectively. Consequently, our results showed that the prediction of the potential geographical distribution of *P. semipunctata* using EM was more reliable than that of using single SDMs.

### 3.2. Potential Geographical Distribution under Near-Current and Future Climates

**Figure 3:**
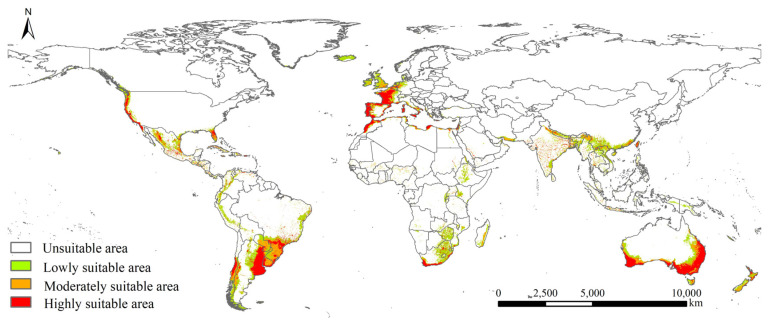
Potential geographical distribution of *Phoracantha semipunctata* under near current climate.

**Figure 4:**
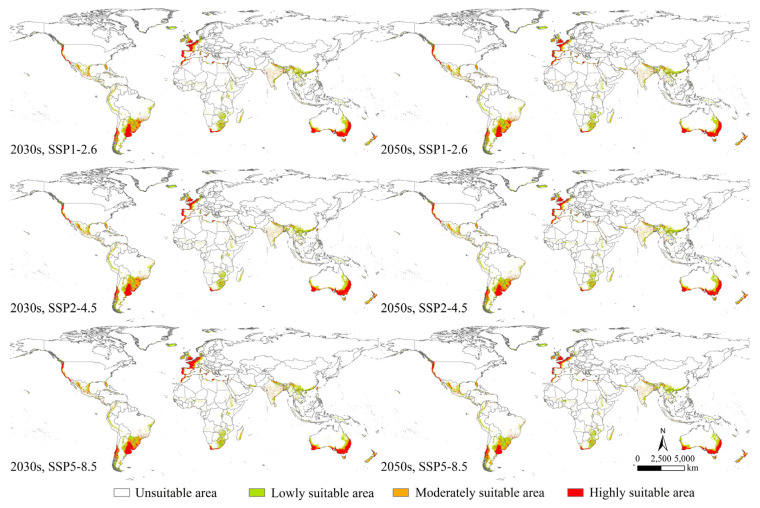
Potential geographical distribution of *Phoracantha semipunctata* under future climate (2030s and 2050s).

The potential geographical distribution of *P. semipunctata* under near-current and future climate covered Asia (China, Laos, Myanmar, Thailand, Cambodia, Vietnam, Malaysia, Indonesia, India, Iran, Pakistan, Israel, Saudi Arabia, United Arab Emirates, and Turkey), Europe (Greece, Italy, Germany, Denmark, the United Kingdom, Belgium, France, Andorra, Spain, Portugal, and Gibraltar), North and Central Americas (the United States, Mexico, Cuba, Panama, Costa Rica, Nicaragua, and Honduras), South America (Colombia, Trinidad and Tobago, Venezuela, Ecuador, Peru, Brazil, Bolivia, Paraguay, Uruguay, Chile, and Argentina), Africa (Morocco, Algeria, Tunisia, Libya, Egypt, Sudan, Kenya, Zambia, Mozambique, Zimbabwe, Botswana, Swaziland, South Africa, Lesotho, and Madagascar), and Oceania (Australia and New Zealand) (Figure 3 and Figure 4).

In the near-current (1970–2000) climate, the total, highly, moderately, and lowly suitable areas of *P. semipunctata* were 1417.35 ×10^4^, 427.18 × 10^4^, 332.28 × 10^4^, and 657.89 × 10^4^ km^2^, respectively (Figure S2). In the 2030s and 2050s, the suitable areas for *P. semipunctata* showed a decreasing trend. 

In the 2030s, under the SSP1-2.6, SSP2-4.5, and SSP5-8.5, the total suitable areas of *P. semipunctata* were 1338.97 × 10^4^, 1327.24 × 10^4^, and 1350.57 × 10^4^ km^2^; the highly suitable areas were 388.42 × 10^4^, 382.05 × 10^4^, and 411.81 × 10^4^ km^2^; the moderately suitable areas were 312.90 × 10^4^, 309.91 × 10^4^, and 297.15 × 10^4^ km^2^; and the lowly suitable areas were 637.65 × 10^4^, 635.28 × 10^4^, and 641.61 × 10^4^ km^2^ (Figure S2), respectively.

In the 2050s, under the SSP1-2.6, SSP2-4.5, and SSP5-8.5 scenarios, the total suitable areas of *P. semipunctata* were 1322.74 × 10^4^, 1301.28 × 10^4^, and 1267.78 × 10^4^ km^2^; highly suitable areas were 376.24 × 10^4^, 374.25 × 10^4^, and 332.55 × 10^4^ km^2^; moderately suitable areas were 306.92 × 10^4^, 289.06 × 10^4^, and 276.47 × 10^4^ km^2^; and low suitable areas were 639.58 × 10^4^, 637.97 × 10^4^, and 658.76 × 10^4^ km^2^ (Figure S2), respectively.

### 3.3. Changes in the Potential Geographical Distribution

**Figure 5:**
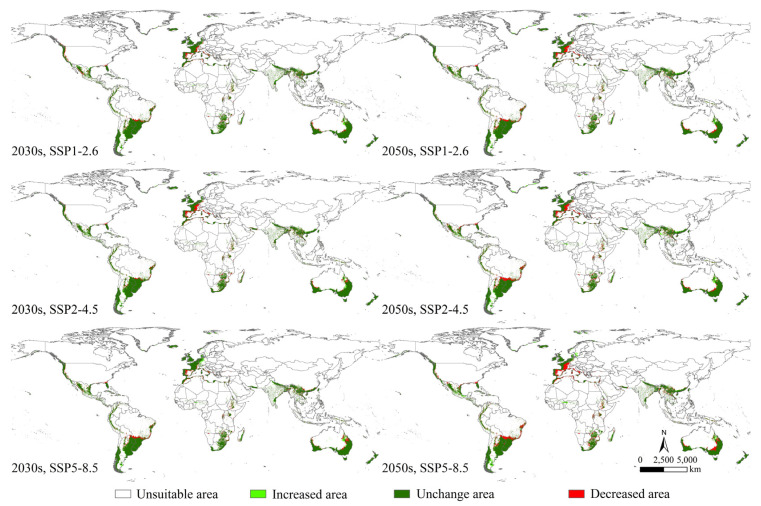
Changes in the potential geographical distribution of *Phoracantha semipunctata* between the near-current and future climates.

Changes in the potential geographical distribution of *P. semipunctata* between the near-current and future climates indicated that the decreased and increased areas of *P. semipunctata* under the SSP5-8.5 scenario of the 2050s were the largest (Figure S2). The areas of increase were primarily located in Europe (France, Italy, Andorra, and Spain), North America (The United States and Mexico), South America (Brazil), Africa (Morocco, Algeria, Tunisia, Ethiopia, and South Africa), and Oceania (Australia) (Figure 5). The areas of decrease were primarily located in Europe (Sweden and Norway), North America (Mexico), South America (Argentina), and Oceania (Australia) (Figure 5). 

In the 2030s, under SSP1-2.6, SSP2-4.5, and SSP5-8.5, the increased areas of *P. semipunctata* were 56.62 × 10^4^, 59.97 × 10^4^, and 78.40 × 10^4^ km^2^, respectively. In 2050s, under SSP1-2.6, SSP2-4.5, and SSP5-8.5 scenarios, the increased areas of *P. semipunctata* were 73.21 × 10^4^, 85.25 × 10^4^, and 102.29 × 10^4^ km^2^, respectively (Figure S2).

In the 2030s, under SSP1-2.6, SSP2-4.5, and SSP5-8.5, the decreased areas of *P. semipunctata* were 131.28 × 10^4^, 145.70 × 10^4^, and 141.13 × 10^4^ km^2^, respectively. In the 2050s, under SSP1-2.6, SSP2-4.5, and SSP5-8.5, the decreased areas of *P. semipunctata* were 164.32 × 10^4^, 197.47 × 10^4^, and 248.09 × 10^4^ km^2^, respectively (Figure S2). 

### 3.4. Distribution Centroids under Near-Current and Future Climates

**Figure 6:**
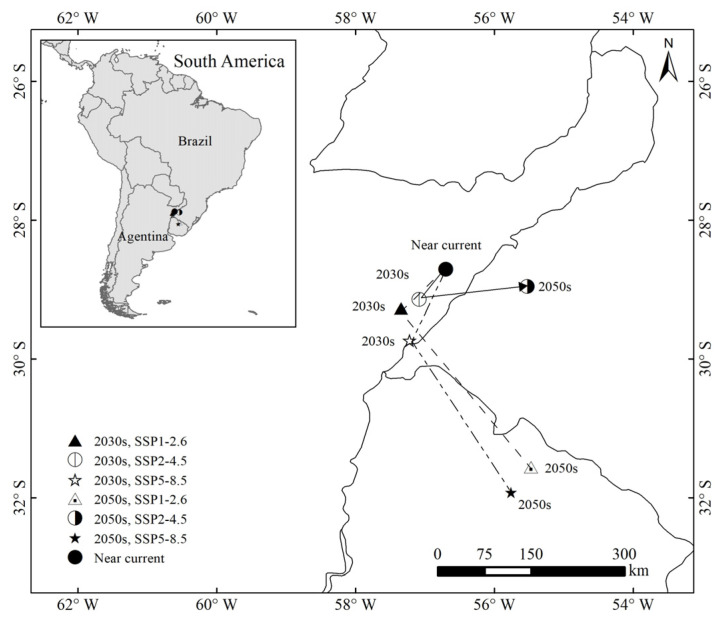
Distribution centroids of *Phoracantha semipunctata* under the near current and future climate scenarios.

From the current time to the 2030s and 2050s, the distribution centroids of *P. semipunctata* were located in Argentina and Brazil and showed a general tendency to shift southward under the three scenarios (SSP1-2.6, SSP2-4.5, and SSP5-8.5) (Figure 6). Under the near-current scenario, the distribution centroid of *P. semipunctata* was located at a particular point (56.68° W, 28.67° S).

In the 2030s and 2050s, under SSP1-2.6, the distribution centroid shifted from its current position to the points (57.34° W, 29.30° S) and (55.50° W, 31.56° S), respectively. Under SSP2-4.5, the distribution centroid shifted from its current position to the points (57.08° W, 29.15° S) and (55.54° W, 28.98° S), respectively; and under SSP5-8.5, it shifted from its current position to the points (57.20° W, 29.74° S) and (51.79° W, 31.89° S), respectively.

### 3.5. Environmental Variables Importance and Response Curve

The temperature and human influence variables were more significant than the others. The top three environmental variables with the highest mean contribution values were the temperature seasonality (bio4, 0.42), human influence index (HII, 0.11), and mean temperature of the warmest quarter (bio10, 0.07). 

The response curves of the significant environmental variables for *P. semipunctata* in the GBM, GLM, and RF models are shown in Figure S3. The suitable range threshold of the environmental variables was classified as a highly suitable area threshold (0.6). With increasing bio4, the presence probability of *P. semipunctata* showed a general downward trend. The suitable range of bio4 for *P. semipunctata* was 0–621, and with an increase in HII, the presence probability of *P. semipunctata* showed an upward trend. The suitable range of the HII for *P. semipunctata* was from 0 to 60. With an increase in bio10, the presence probability of *P. semipunctata* first decreased and then increased. The suitable range of bio10 for *P. semipunctata* was between 7–33 °C. 

### 3.6. Environmental Variables Importance and Response Curve

**Figure 7:**
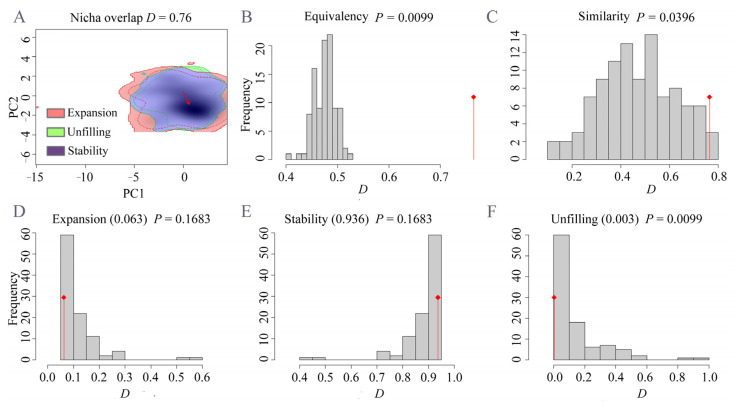
Niche comparison for *Phoracantha semipunctata* between native (Australia) and invasive areas. (**A**): niche overlap on two principal component axes (PC1 and PC2). (**B**,**C**): equivalency and similarity tests. (**D**–**F**): niche expansion, stability, and unfilling tests. Red lines in the plots indicate the observed value of niche overlap, and gray bars indicate the distribution of D values for 100 simulated comparisons.

Niche comparison tests a high degree of niche overlap (Schoener’s D = 0.78) (Figure 7A). The equivalency and similarity tests indicated that the two niches were not equivalent but were more similar than would be expected by chance (equivalency test, *p* = 0.0099, similarity test, *p* = 0.0396) (Figure 7B,C). The expansion, stability, and unfilling indices and tests revealed that most of the simulated environments were occupied by the stability area (0.936), and a small part of the simulated environments was occupied by the small expansion (0.063) and unfilling (0.003) areas (Figure 7A,D–F). Consequently, the niche of *P. semipunctata* was conservative and stable during the invasion process.

### 3.7. Environmental Variables Importance and Response Curve

**Figure 8:**
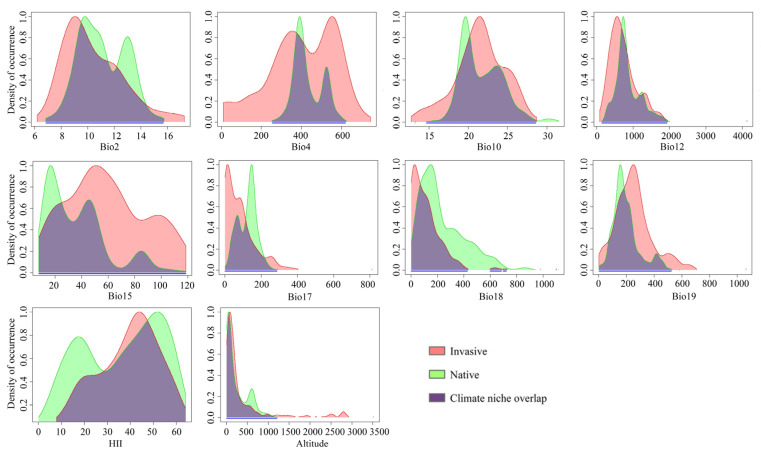
Predicted niche occupancy profiles. Bio2: mean diurnal range. Bio4: temperature seasonality. Bio10: mean temperature of the warmest quarter. Bio12: annual precipitation. Bio15: precipitation seasonality. Bio17: precipitation of driest quarter. Bio18: precipitation of warmest quarter. Bio19: precipitation of coldest quarter. HII: human influence index.

Our predicted niche occupancy profiles revealed heterogeneity in the environmental requirements of *P. semipunctata* populations in native and invasive areas (Figure 8). Regarding temperature requirements, including the mean diurnal range (bio2), temperature seasonality (bio4), and mean temperature of the warmest quarter (bio10), invasive populations of *P. semipunctata* were more suitable than native populations for wider temperature ranges. Regarding precipitation, human influence, and topography requirements, including annual precipitation (bio12), precipitation seasonality (bio15), precipitation of driest quarter (bio17), precipitation of warmest quarter (bio18), precipitation of coldest quarter (bio19), human influence index (HII), and altitude, the invasive and native populations were not significantly different. Consequently, if records of *P. semipunctata* are observed in those environments in the native area, they are also likely to be observed in the invasive areas.

## 4. Discussion

### 4.1. Model Prediction Significance

*Phoracantha semipunctata* has spread and established populations on six continents in which eucalypts have been planted [18]. Consequently, estimating its global distribution pattern is the basis for early warning, prevention, control, and management. To our knowledge, the present study is the first to select an EM to predict the potential global geographical distribution of *P. semipunctata*. SDMs have been frequently selected to predict the potential geographical distribution of invasive alien insects. Previous studies have revealed the superior predictive performance of EM over a single SDM, and single model uncertainty can be avoided or reduced by EM [40,41]. For instance, some studies on the prediction of the distribution pattern of *Vespa velutina* in the Mediterranean island regions [42], estimating the global invasion risk for *Hemiculter leucisculus* [43] and predicting the habitat suitability for the invasive bee in Hawai’i [44], indicated that the predictions using an EM were more reliable than those using a single SDM. We also assessed niche dynamics during the global invasion process and significant variables affecting the global distribution pattern of *P. semipunctata*. Our results provide a reference for the early warning, prevention, and control of *P. semipunctata*.

### 4.2. Suitable Environmental Conditions

Temperature is an important factor that affects both the developmental time and fecundity of insects [45,46]. Our findings indicated that temperature seasonality and the mean temperature of the warmest quarter were significant temperature variables affecting the global distribution pattern of *P. semipunctata*. The egg masses of *P. semipunctata* were typically found during the warm quarter, while the developmental period of *P. semipunctata* pupae averaged 32–40 days during early spring to late summer, with an average of approximately 110 days in autumn [18]. The thermic integral of *P. semipunctata* needed to complete one generation as 1510 degree-days with the minimum threshold temperature (11.5 °C) [47]. These studies indicate that warm climatic conditions favor the development of *P. semipunctata* populations, and that the temperature of the warmest quarter may have a great impact on the distribution and survival of *P. semipunctata*. Mediterranean climate (dry and hot summer) allows more generations of *P. semipunctata* per year than in other climates [47]. Our results showed that suitable areas for *P. semipunctata* were located in the Mediterranean and coastal ranges worldwide. Consequently, the above-mentioned findings indicate that the environmental conditions of the Mediterranean and coastal ranges are more suitable for *P. semipunctata*. Human activities have facilitated the global biological invasion process, from disrupting ecosystems to transporting invasive alien species [48,49,50]. Our results show that with an increase in the HII, the presence probability of *P. semipunctata* showed an upward trend. The host plants of *P. semipunctata* are primarily Eucalyptus species, which are one of the most economically important trees and are widely planted worldwide [51]. The main spread pathways are human-mediated transport of Eucalyptus wood worldwide [16]. Consequently, human influences play a key role in the distribution and survival of *P. semipunctata* worldwide. 

### 4.3. Niche Dynamic and Prospective Distribution Changes

Niche conservatism of the IAS is considered common in different geographical locations [52,53]. Species arriving in zones with environmental conditions similar to those of their native zones are more likely to successfully establish populations [11]. In the present study, we assessed niche dynamics during the global invasion of *P. semipunctata*. The degree of niche overlap between the native and invasive areas was high, further indicating that *P. semipunctata* occupies environmental niches in invasive areas that are similar to their native areas. Previous studies have compiled ample evidence of niche conservatism of the IAS during the invasion process at biogeographic scales. For instance, most of the 434 IAS largely conserved their climatic niche between native and invasive ranges [54]. Collectively, our results support the niche conservatism hypothesis.

To date, *P. semipunctata* is primarily found in Asia (Israel), Europe (Italy, Spain, Portugal, France, and the Netherlands), North America (the United States and Mexico), South America (Argentina, Chile, Peru, Uruguay, Brazil, and Bolivia), Africa (South Africa, Tunisia, Algeria, Zambia, Egypt, Mauritius, Swaziland, Morocco, Mozambique, Malawi, Lesotho, Zimbabwe, and Ethiopia), and Oceania (Australia and New Zealand). Our results indicated that the potentially invasive areas were primarily located in Asia (China, India, Laos, Myanmar, Thailand, Cambodia, Vietnam, Malaysia, Indonesia, Iran, Pakistan, Saudi Arabia, United Arab Emirates, and Turkey) and Europe (Greece, Germany, Denmark, the United Kingdom, Belgium, Andorra, and Gibraltar), where wild populations of *P. semipunctata* have not yet been found. The above countries were planting eucalyptus or have industries based on eucalyptus wood that would be affected by *P. semipunctata*. More attention should be paid to early warning and monitoring of *P. semipunctata* in potentially invasive areas. For the early warning and monitoring measure of *P. semipunctata*, the freshly cut trap logs that are treated with pesticide can be placed in woodlots to attract adult beetles [18]. Suitable environmental conditions play a key role in the degree of risk posed by *P. semipunctata*, which may influence eucalyptus trees. The main global spread pathways are via the human-mediated transport of eucalyptus wood [16]. Consequently, on a global scale, for the areas where wild populations of *P. semipunctata* have not yet been found, more attention is required concerning the eucalyptus wood trade with other countries, especially where *P. semipunctata* has established naturalized populations. Once the wild population of *P. semipunctata* is found, it should be rapidly eradicated with cultural and biological control measures. For instance, silvicultural measures are the most effective control measures in Spain and Morocco, primarily through the selection of appropriate trees for each zone [55]. Parasitoids, including *Platystasius transversus* and *Avetianella longoi*, are the most effective natural enemies of *P. semipunctata* [56,57].

Climate change and human activities have altered the distribution pattern of species, including the shrinking and expansion of their geographical distribution [58,59]. A previous study showed that losses to unmanaged pests may decrease when climate change causes less favorable environmental conditions for the establishment of pest populations [60]. Some case studies have shown that climate change decreases the geographical distribution of IAFPs. For instance, climate warming has globally resulted in a decrease in suitable areas for *Dalbulus maidis* [61]. The geographical distribution of the vine mealybug (*Planococcus ficus*) is expected to decrease in future climates [58]. Climate change has shifted the distribution centroid of species poleward. In our study, the distribution centroid of *P. semipunctata* showed a general tendency to shift southward under climate change conditions. Our results support the hypothesis that climate-warming-driven species shift poleward and decrease in areas where climate warming causes less suitable environmental conditions for species establishment. 

In summary, we are the first attempt to present a novel framework for integrating the SDMs and niche dynamic into the estimation of potentially suitable areas for *P. semipunctata* worldwide and niche comparison between the native and invasive areas. Our results can guide the worldwide selection of planted areas for eucalyptus species without the potential invasion risk of *P. semipunctata*, and provide insights into the effects of climate change on the global distribution pattern of *P. semipunctata*. Finally, the results can also serve as a reference for the early warning, prevention, control, and management of *P. semipunctata* during future invasions.

## 5. Conclusions

Based on the global occurrence records of *P. semipunctata* and related environmental variables, we constructed an EM using the three most accurate single SDMs to predict the potential geographical distribution worldwide. We also simulated the niche dynamics of *P. semipunctata* during the global invasion process to assess invasion risk in the future. First, the potential global geographical distribution of *P. semipunctata* is primarily located in western and southwestern Asia, western Europe, western and southern North America, southern South America, southern Africa, and eastern and southern Oceania. The potential invasive zones (wild populations of *P. semipunctata* have not yet been found) were primarily located in eastern Asia and eastern Europe. Second, *P. semipunctata* largely conserves its niche during the invasion process. Finally, the distribution pattern was primarily attributed to temperature and human influence variables. The early warning of biological invasions is commonly considered the most effective measure for the management of invasive alien species. Consequently, countries should strictly implement plant quarantine regulations for imported eucalyptus logs to prevent the invasion of *P. semipunctata*. In the future, we will focus on the modeling integration for SDMs, evolutionary analysis, and phylodynamic inference for *P. semipunctata* so as to better reconstruct its distribution, origin, and spread. 

## Data Availability

The data presented in this study are available in this article.

## Supplementary Materials

The following information is available online at https://www.mdpi.com/article/10.3390/insects14010084/s1, Figure S1: Pearson correlation coefficients of 19 bioclimatic variables for four study species; Figure S2: The areas of potential geographical distribution and changes of *Phoracantha semipunctata* under near-current and future climate (the 2030s and 2050s); Figure S3: The areas of potential geographical distribution and changes of *Phoracantha semipunctata* under near-current and future climate (the 2030s and 2050s); Table S1: All environmental variables; Table S2: Environmental variables related to the distribution of *Phoracantha semipunctata*.
