# Supplementary material for: Constructing an Ensemble Model and Niche Comparison for the Management Planning of Eucalyptus Longhorned Borer Phoracantha semipunctata under Climate Change
Source: Insects. 2023 Jan 13;14(1):84. doi: 10.3390/insects14010084 (PMC9866156; doi:10.3390/insects14010084)
Supplement: Supplementary file 1 [file insects-14-00084-s001.zip › insects-2089830-supplementary.pdf]

Supplementary Material

1 Supplementary Figures and Tables

1.1 Supplementary Figures

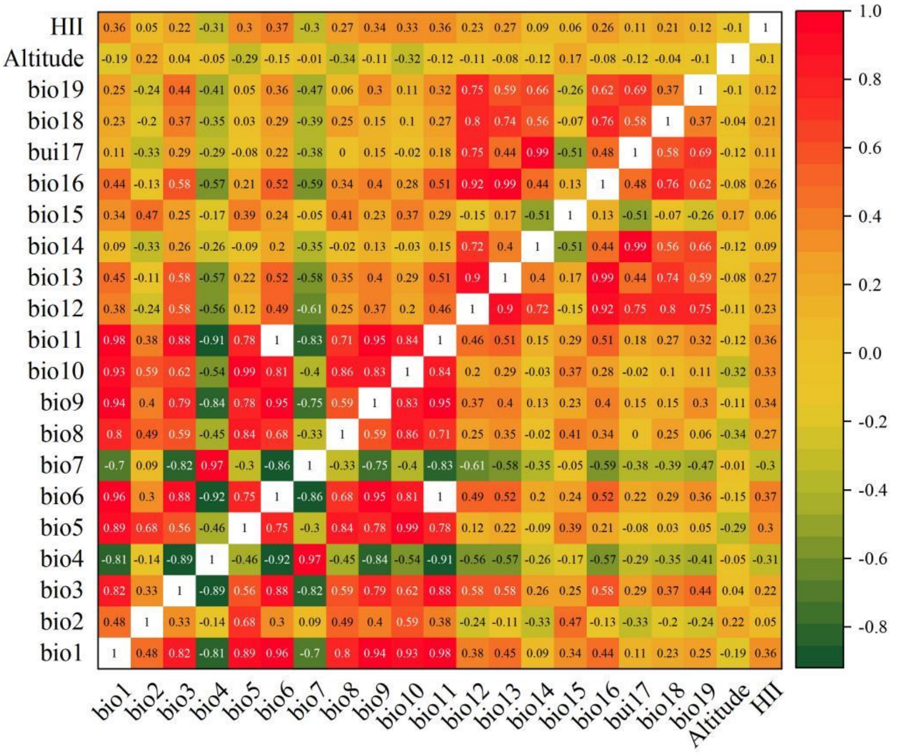

Figure S1 Pearson correlation coefficients of 19 bioclimatic variables for four study species.

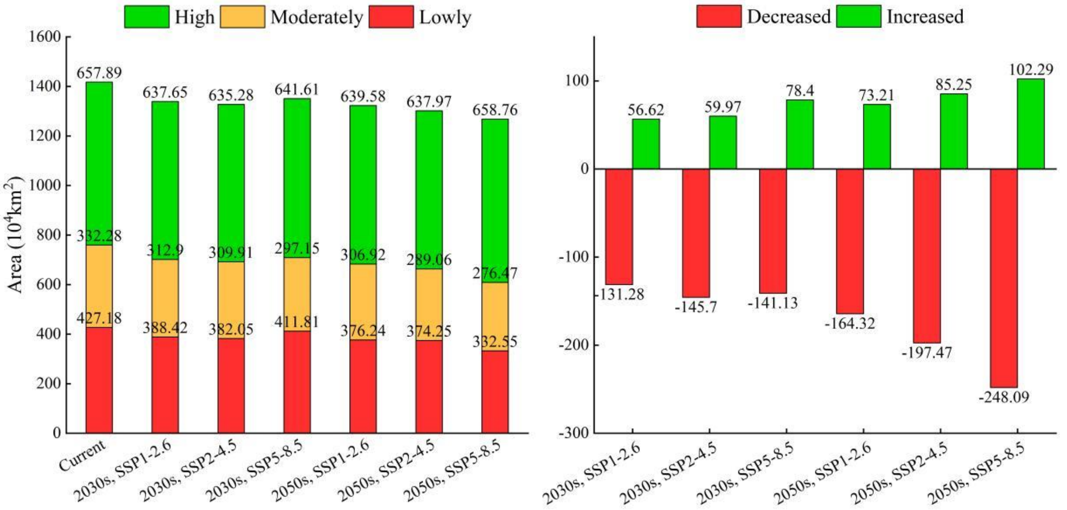

Figure S2 The areas of potential geographical distribution and changes of *Phoracantha semipunctata* under near current and future climate (2030s and 2050s)

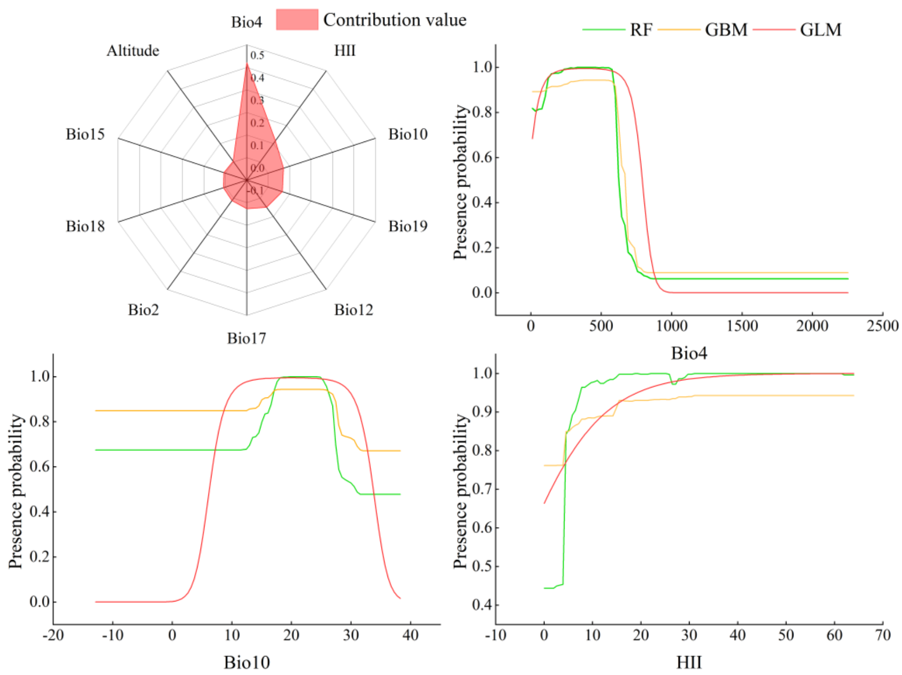

Figure S3 The areas of potential geographical distribution and changes of *Phoracantha semipunctata* under near current and future climate (2030s and 2050s)

1.2 Supplementary Tables

Supplementary Table S1 All environmental variables

Variables	Description	Unit	Resolution	Sources
Bio1	Annual mean temperature	℃	2.5 arc minutes	WorldClim
Bio2	Mean diurnal range	℃	2.5 arc minutes	WorldClim
Bio3	Isothermality	-	2.5 arc minutes	WorldClim
Bio4	Temperature seasonality	℃	2.5 arc minutes	WorldClim
Bio5	Max temperature of warmest month	℃	2.5 arc minutes	WorldClim
Bio6	Min temperature of coldest month	℃	2.5 arc minutes	WorldClim
Bio7	Temperature annual range	℃	2.5 arc minutes	WorldClim
Bio8	Mean temperature of wettest quarter	℃	2.5 arc minutes	WorldClim
Bio9	Mean temperature of driest quarter	℃	2.5 arc minutes	WorldClim
Bio10	Mean temperature of warmest quarter	℃	2.5 arc minutes	WorldClim
Bio11	Mean temperature of coldest quarter	℃	2.5 arc minutes	WorldClim
Bio12	Annual precipitation	mm	2.5 arc minutes	WorldClim
Bio13	Precipitation of wettest month	mm	2.5 arc minutes	WorldClim

Bio14	Precipitation of driest month	mm	2.5 arc minutes	WorldClim
Bio15	Precipitation seasonality	-	2.5 arc minutes	WorldClim
Bio16	Precipitation of wettest quarter	mm	2.5 arc minutes	WorldClim
Bio17	Precipitation of driest quarter	mm	2.5 arc minutes	WorldClim
Bio18	Precipitation of warmest quarter	mm	2.5 arc minutes	WorldClim
Bio19	Precipitation of coldest quarter	mm	2.5 arc minutes	WorldClim
Altitude	Altitude	m	2.5 arc minutes	WorldClim
HII	Human Influence Index	-	2.5 arc minutes	NASA Socioeconomic Data and Applications Center

Supplementary Table S2 Environmental variables related to the distribution of *Phoracantha semipunctata*

Variables	Description	Unit
Bio2	Mean diurnal range	℃
Bio4	Temperature seasonality	℃
Bio10	Mean temperature of warmest quarter	℃
Bio12	Annual precipitation	mm
Bio15	Precipitation seasonality	
Bio17	Precipitation of driest quarter	mm
Bio18	Precipitation of warmest quarter	mm
Bio19	Precipitation of coldest quarter	mm
Altitude	Altitude	m
HII	Human Influence Index	-
